# Recent Advances in Molecularly Imprinted Membranes: Structure–Activity Relationships, Morphology Control, and Separation Applications

**DOI:** 10.3390/molecules31142479

**Published:** 2026-07-15

**Authors:** Xuanxu Shi, Jiaqi Jiang, Wanqi Du, Maobin Wei, Minjia Meng

**Affiliations:** 1 College of Physics, Jilin Normal University, 1301 Haifeng Street, Siping 136000, China; 2 School of Chemistry and Chemical Engineering, Jiangsu University, Zhenjiang 212013, China; 3 Pharmaceutical Sciences Laboratory and Turku Bioscience Center, Åbo Akademi University, 20520 Turku, Finland

**Keywords:** molecularly imprinted membrane, selective separation, physical properties, morphology optimization

## Abstract

Molecularly imprinted membranes (MIMs) have demonstrated tremendous potential in the field of high-efficiency separation due to their specific molecular recognition capabilities. This review aims to elucidate the underlying mechanisms governing MIMs’ performance and, moving beyond traditional classification frameworks, systematically reconstructs the classification system for MIMs from the perspectives of the spatial distribution of imprinted sites, the chemical topology of the matrix, and mass transfer kinetics. The article focuses on the decisive influence of key physical parameters such as pore size, specific surface area, hydrophilicity/hydrophobicity, and swellability on separation efficiency. It provides an in-depth analysis of the spatial matching between pore size and target molecules, the nonlinear relationship between specific surface area and adsorption capacity, and the mechanisms by which mechanical strength and swelling behavior constrain the long-term stability of the membranes. Addressing the common bottlenecks faced by MIMs “high mass transfer resistance and poor accessibility of recognition sites” this paper critically summarizes cutting-edge morphological optimization strategies, such as multi-level pore construction, nanocomposite reinforcement, and surface topological engineering, aiming to elucidate how microstructural regulation can achieve a synergistic enhancement of both high throughput and high selectivity. Finally, by reviewing breakthroughs in MIMs applications for biomedical extraction and environmental pollutant remediation, this review not only clarifies the principles governing material suitability across different scenarios but also provides a systematic technical reference for the development of next-generation, high-performance, industrial-scale MIMs.

## 1. Introduction

Separation science [1,2,3] is a core foundational discipline dedicated to the efficient identification, extraction, enrichment, and deep purification of target substances from complex systems characterized by multiple components, strong interference, and highly similar structures. Based on the physical and chemical differences between substances, it constructs highly efficient separation systems to achieve precise identification, capture, and controlled elution of target molecules, thereby fundamentally eliminating the effects of matrix interference and structurally similar impurities [4,5,6,7]. Separation science not only underpins breakthroughs in fundamental research across numerous fields (including chemical synthesis [8], materials preparation [9,10], bioanalysis [11,12,13,14], pharmaceutical R&D [15,16], environmental monitoring [17,18,19], and energy conversion [20]) but also serves as the core enabling and bottleneck technology for translating laboratory achievements into industrial, large-scale, and high-end production in these sectors. Whether it involves screening for active compounds in complex natural products, preparing high-purity active pharmaceutical ingredients and biomolecules for biopharmaceuticals, conducting precise detection of ultra-trace pollutants in environmental systems, or scaling up the production of ultra-high-purity chemicals and critical materials for the new energy and electronics industries, all rely heavily on separation science to provide reliable technical assurance.

In summary, without modern separation technologies capable of high selectivity, high throughput, deep separation, and high-purity purification, it would be impossible to obtain high-purity substances that meet the demands of high-end applications, to achieve precise and reliable analytical testing, or to support the efficient, stable, and sustainable large-scale production of modern industry.

Traditional separation technologies primarily rely on differences in the physicochemical properties of substances, with core methods including distillation [21], extraction [22], crystallization [23], precipitation [24], adsorption [25], and membrane separation [26,27]. As a classic separation method, distillation utilizes the differences in volatility (or boiling points) of components in a liquid mixture to achieve separation and purification through the processes of heating, vaporization, and condensation. It is widely applied in fields such as petrochemicals [28] and solvent recovery [29]. Extraction technology, on the other hand, exploits the differences in solubility of the target substance in two immiscible (or slightly soluble) solvents, causing it to transfer from one phase to another, thereby achieving separation. It plays a crucial role in natural product extraction [30], hydrometallurgy [31], and wastewater treatment [32]. Both crystallization and precipitation involve obtaining solid-phase products from the liquid phase. Crystallization involves controlling conditions such as temperature and solvent to bring a solution to a supersaturated state, causing the target substance to precipitate as high-purity crystals [33]; it is a key method for obtaining high-purity solid substances. Precipitation, on the other hand, typically involves chemical reactions or changes in physical conditions (such as pH or temperature) to form insoluble solids from specific components in a solution, and is commonly used for the removal or preliminary enrichment of ions [34]. Adsorption is a core technology that utilizes the surface of a solid to selectively attach specific components in a fluid (gas or liquid), thereby achieving the separation and purification of substances [35].

Membrane separation, as a relatively modern technology, utilizes membranes with selective permeability to separate, purify, and concentrate components in a mixture under driving forces such as pressure gradients, concentration gradients, or potential gradients. Based on pore size and separation mechanisms, membrane separation is primarily categorized into microfiltration [36,37], ultrafiltration [38,39], nanofiltration [40], and reverse osmosis [41], demonstrating significant potential in water treatment, biopharmaceuticals, and the food industry.

Although these conventional separation methods have been widely adopted in industrial production, their inherent limitations have become increasingly apparent when faced with increasingly complex separation systems and higher purification standards. For example, distillation processes are typically associated with high energy consumption; extraction techniques rely on large amounts of organic solvents, which are not only costly but may also lead to environmental pollution; crystallization and precipitation methods face challenges in product selectivity, purity control, and yield; while membrane separation technologies may encounter issues such as membrane fouling, flux decline, and insufficient selectivity. Therefore, developing novel separation technologies that combine high selectivity, high flux, low energy consumption, and environmental friendliness or innovatively coupling and optimizing existing processes has become a core issue urgently requiring resolution in the field of separation science, as well as an inevitable requirement for driving technological upgrades and sustainable development in related industries [42,43,44].

Molecularly imprinted membranes (MIMs) are particularly well-suited for the separation of structural analogues to achieve high-purity substances. Molecularly imprinted membranes [45] are a new type of functional separation material that combines molecular imprinting technology [46,47,48,49,50] with membrane separation technology [51]. Using the target molecule as a template, functional monomers are pre-assembled with the template molecule through covalent or non-covalent interactions during membrane preparation or functionalization, followed by processes such as polymerization, cross-linking, and phase transformation to form a stable three-dimensional network membrane structure [52,53]; Subsequently, the template molecules are removed through elution, ultimately leaving behind specific recognition cavities within the membrane matrix that are highly complementary to the target molecule in terms of three-dimensional structure, functional group distribution, and binding sites. These specific recognition sites can achieve highly selective recognition and targeted capture of the target molecule, much like an “antigen–antibody” interaction [54]. Although molecularly imprinted membranes possess high selectivity for recognition, they also have certain limitations. First, it is difficult to precisely control the uniformity of the imprinting sites on the membrane, which leads to an uneven distribution of recognition sites and consequently affects performance in practical applications. Second, the mechanical strength and stability of the membrane are constrained by the inherent properties of the material; it is prone to degradation or deformation under harsh conditions such as high temperatures and strong acids or bases, resulting in insufficient durability [55]. Furthermore, existing preparation processes are complex and costly, and functionalization is challenging, making it difficult to meet the complex demands of diverse application scenarios. Finally, the recognition efficiency of molecularly imprinted membranes for target analytes in complex matrices is easily affected by interfering substances; coupled with slow binding kinetics, this results in insufficient real-time detection capabilities. Therefore, there is an urgent need to overcome these bottlenecks through material optimization and the integration of new technologies [56].

However, through the membrane’s inherent pore structure and size-exclusion effects, the target molecules undergo size-based screening and mass-transfer regulation at the molecular level. The synergistic action of these two mechanisms overcomes the shortcomings of traditional membrane separation, which relies solely on size differences and suffers from poor selectivity while also addressing the issues of high mass transfer resistance and slow separation rates associated with conventional molecularly imprinted materials. Consequently, even under the interference of complex systems, the selective separation, enrichment, and purification of target molecules can be achieved efficiently and specifically, providing a novel technical pathway for highly selective and precise separation [57,58,59].

The performance of molecularly imprinted membranes varies significantly; for the same separation medium, molecularly imprinted membranes prepared on different matrix substrates exhibit distinct separation effects. For example, organic polymer matrices, such as polyvinylidene fluoride (PVDF)-based molecularly imprinted membranes [60], feature high mechanical strength, solvent resistance, and strong hydrophobicity, making them suitable for organic solvent systems and industrial-scale preliminary separation; Natural polymer matrices, such as cellulose (NC) [5], exhibit excellent hydrophilicity, biocompatibility, and degradability. They are suitable for the detection of biological samples and food in pure aqueous systems; inorganic matrices [61], such as silica (SiO_2_)-based molecularly imprinted membranes, feature high rigidity, good stability, and resistance to swelling, making them suitable for chiral separation and the preparation of high-purity substances; Organic-inorganic hybrid matrices [62], such as organic polymer-MOF hybrid molecularly imprinted membranes, feature multi-level pore structures and high specific surface areas, making them suitable for deep separation; functionalized matrices [63], such as magnetic molecularly imprinted membranes, offer magnetic separation capabilities and ease of recovery, making them suitable for complex matrices and rapid separation and elution. However, the relationship between different separation scenarios and membrane suitability has not yet been fully elucidated by researchers, resulting in a scarcity of reports on the performance patterns of molecularly imprinted membranes in selective separation. Therefore, the purpose of this review is to clarify the applicability patterns of molecularly imprinted membranes in various separation scenarios.

## 2. Classification of MIMs

MIMs can be classified based on the formation mechanism of imprinted sites, the chemical composition of the membrane matrix, and the mass transfer mechanism involved in separation. Table 1 shows a multidimensional separation system. This multidimensional classification system facilitates a deeper understanding of the structural characteristics and functional differences among various molecularly imprinted membranes.

### 2.1. Classification by Imprint Site Formation Method

**Figure 1:**
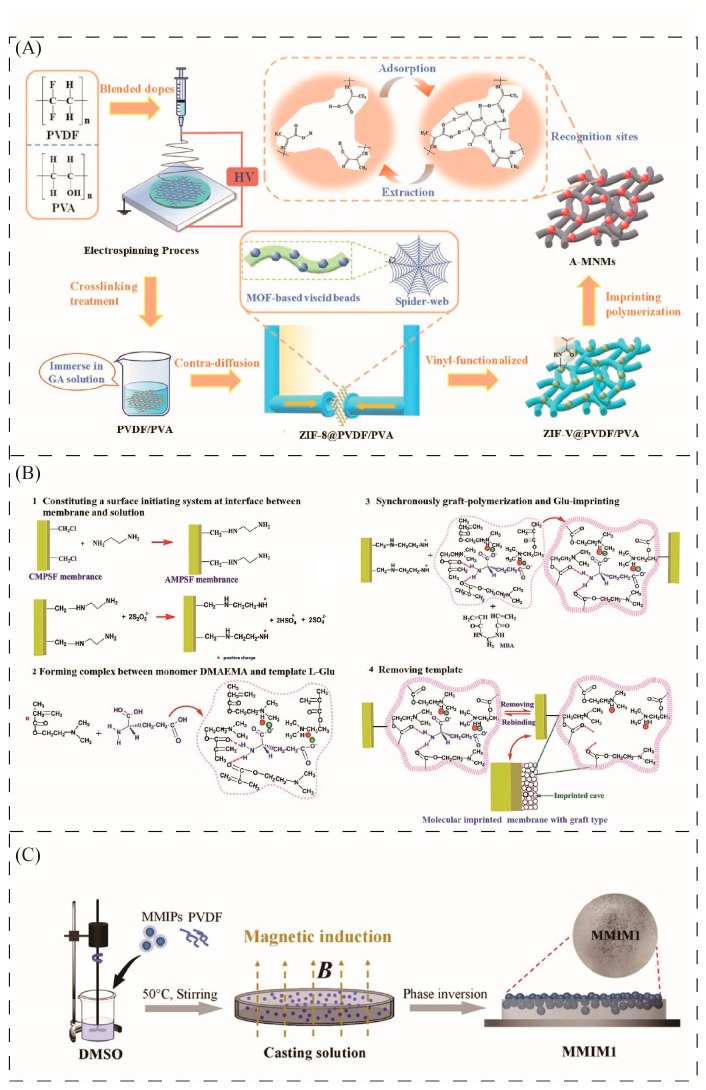
(**A**) In situ polymerization on a PVDF/PVA blended nanofiber membrane [65]. Copyright © 2021, American Chemical Society (**B**) Graft/cross-linking polymerization on an AMPSF microfiltration membrane [67]. © Society of Chemical Industry (**C**) Blending a magnetically imprinted polymer with PVDF and converting the mixture into a membrane [68]. © 2020 Elsevier B.V. All rights reserved.

Based on the spatial distribution of imprinted sites within the membrane, molecularly imprinted membranes can be classified into bulk-imprinted membranes, surface-imprinted membranes, and composite membranes. Figure 1 shows a schematic diagram of the distribution of these three different imprinting sites.

#### 2.1.1. Bulk-Imprinted Membranes

Whole-film molecularly imprinted membranes are formed through in situ polymerization [75,76]. They are functional separation membranes featuring a continuous, non-porous or porous monolithic framework, with specific recognition sites uniformly embedded within the membrane matrix. A common synthesis method involves mixing template molecules, functional monomers, crosslinkers, porogens, and solvents in specific proportions to form a stable, homogeneous casting solution. This solution is then poured into a mold or coated onto a support material, and the polymerization reaction is initiated via thermal or photo-initiation. After polymerization, the template molecules are eluted to obtain the molecularly imprinted membrane [77,78]. Sergeyeva et al. [64] prepared molecularly imprinted membranes using an in situ polymerization method; however, highly cross-linked materials resulted in membranes with unstable physical properties. To address this limitation, researchers introduced various regulators and pore-forming agents to improve the physical properties of the membranes. Experimental results demonstrated that the introduction of these additives significantly enhanced the mechanical stability, flexibility, and flux of the membranes. Building on this, Xing et al. [65] performed in situ polymerization on a vinyl-functionalized nanofiber membrane doped with Metal–Organic Framework (ZIF-V@PVDF/PVA); the high specific surface area provided by the nanofiber substrate, which mimics adhesive beads, facilitates the formation of more imprinting recognition sites during the imprinting process.

#### 2.1.2. Surface-Imprinted Membranes

Unlike bulk-imprinted membranes, surface-imprinted membranes are functional separation membranes in which a thin, specific recognition layer is formed on the surface of a support substrate through template-induced polymerization, with recognition sites distributed only on or near the membrane surface [79,80]. The process begins with surface activation of the base membrane to introduce polymerizable sites, followed by in situ graft polymerization of the template, functional monomers, crosslinkers, and initiators on the surface to form a thin imprinted layer [81,82]. For example, Li et al. [66] reported a surface molecularly imprinted membrane for chiral separation by coating cellulose acetate onto a zirconia-modified alumina nanochannel membrane. The results indicated that this nanochannel membrane exhibits higher flux and selectivity compared to conventional membranes, with the two properties exhibiting a trade-off relationship. Furthermore, Gao et al. [67] performed surface polymerization on aminated polysulfone microfiltration membranes to effectively separate L-Glu; the grafted imprint cross-linked network exhibited specific recognition and selective permeability toward L-Glu, while the aminated polysulfone microfiltration membrane provided high flux and excellent mechanical properties.

#### 2.1.3. Composite Membranes

Composite membranes are functional separation membranes in which pre-prepared molecularly imprinted materials are uniformly mixed with membrane matrix materials in a specific ratio and prepared via film-forming processes such as cast film formation or phase inversion [83,84]. The imprinted polymer system is directly introduced into the membrane casting solution (e.g., PVDF, Polyethersulfone, etc.), and composite membranes are prepared using the phase inversion method. This physical blending strategy is not only simple and cost-effective but also promotes the uniform dispersion of the imprinted material within the matrix; the resulting widely distributed recognition sites effectively enhance the membrane material’s adsorption capacity for target molecules. Notably, by adjusting the addition ratio and particle size of the imprinted material, the membrane’s selectivity, flux, and mechanical stability can be flexibly tuned [85,86]. For example, Bai et al. [68] reported a magnetically guided preparation method: first, magnetic molecularly imprinted particles were incorporated into PVDF to form a casting solution; then, magnetic forces were used to attract the particles to the upper surface of the solution; Finally, the membrane was formed through liquid nitrogen freezing and a phase inversion process. This strategy results in a higher concentration of imprinted sites on the membrane surface. Experimental results indicate that, compared to imprinting membranes prepared by conventional blending methods, this membrane exhibits superior dynamic separation performance. Furthermore, Xing et al. [69] innovatively introduced MOF particles into the solidification bath during the film-forming process and utilized light irradiation to drive the self-assembly of the membrane. Studies have shown that self-assembled MOFs enhance specific rebinding capacity and promote delayed permeation of target molecules. More importantly, the photocatalytic properties of MOF endow this molecularly imprinted membrane with rapid regeneration capabilities: light-induced reactive oxygen species (ROS) can effectively degrade template molecules adsorbed at the molecularly imprinted sites, thereby ensuring the membrane maintains long-lasting selective separation performance.

### 2.2. Classification by Membrane Matrix

Based on the chemical nature of the membrane matrix, molecularly imprinted membranes can be classified into three major categories: organic polymer-based, inorganic-based, and hybrid material-based.

#### 2.2.1. Organic Polymer-Based MIMs

This class of membranes is primarily composed of organic polymeric materials, using organic polymers [87,88] (such as acrylates, polystyrene, polysulfone, PVDF, etc.) or natural polymers [89,90] (chitosan, cellulose, alginate) as the base membrane. Through molecular imprinting technology, the membrane is engineered to contain imprinted sites and pores. For example, polymerization occurs on the surface or within the pores of the membrane. By combining template molecules, functional monomers, crosslinking agents, and porogens, the template molecules and functional monomers are pre-assembled in a specific ratio. This mixture is then combined with an organic polymer matrix to form a stable casting solution. Subsequently, film-forming processes such as phase inversion, in situ polymerization, or melt spinning are employed to convert the casting solution into a membrane with a specific structure; after the template molecules are removed by elution, a molecularly imprinted separation membrane is formed [91,92]. For example, Segundo et al. [70] reported a method for preparing molecularly imprinted membranes based on electrospinning technology. They first loaded a polymer solution containing polycaprolactone (PCL) into an electrospinning syringe, then systematically optimized key spinning parameters, including needle diameter, flow rate, applied voltage, and working distance. By precisely controlling these parameters, they successfully prepared molecularly imprinted membranes with regular morphology and uniform size distribution. Maria et al. [71] reported an organic-based molecularly imprinted membrane using chitosan as the matrix. This membrane, prepared via in situ polymerization, exhibited highly selective binding capacity toward phenolic pollutants in water. The introduction of the chitosan matrix not only conferred good biodegradability to the membrane but also significantly reduced production costs.

#### 2.2.2. Inorganic-Based MIMs

Inorganic-based molecularly imprinted membranes use inorganic materials such as ceramics, SiO_2_, and TiO_2_ as core matrices or carriers [93,94]. These functional separation membranes contain specific recognition sites that match the target molecules and are prepared using molecular imprinting technology, forming an “inorganic support + imprint” structure. The preparation process begins by selecting an inorganic material as the matrix, followed by surface activation through hydroxylation, silanization, or similar treatments to enhance the binding affinity with the imprinting system. Subsequently, template molecules, functional monomers compatible with the inorganic matrix, cross-linking agents, and solvents are mixed in specific proportions. The functional monomers and template molecules pre-assemble into complexes via coordination bonds, hydrogen bonds, and other interactions. Through methods such as sol–gel or in situ polymerization, the imprinting sites are uniformly distributed on the surface or within the pores of the inorganic matrix, forming a membrane with a porous structure. Finally, elution is performed to produce the molecularly imprinted separation membrane. For example, Bagheri et al. [72] reported a molecularly imprinted membrane in which magnetic polyaniline and graphene oxide (GO) were dispersed in ultrapure water and subjected to ultrasonic treatment, yielding the M@PANI/rGO material. Building on this, a highly selective molecularly imprinted membrane was successfully constructed by performing the electropolymerization of pyrrole on the surface of a magnetic electro-paste electrode; this method has been successfully applied to the detection and separation of rutin.

#### 2.2.3. Hybrid Material-Based MIMs

Hybrid material-based molecularly imprinted membranes use organic polymers as the main backbone and incorporate inorganic nano-functional phases as functional units, forming a composite structure of “organic support and inorganic functional phase and imprint” [95,96]. The general synthesis method involves adding template molecules, functional monomers, and crosslinkers to a solvent in specific proportions, achieving template-monomer pre-assembly via hydrogen bonds or coordination bonds. Activated inorganic components are then added to the organic matrix material to form a casting solution. Through in situ polymerization, phase transformation, and other processes, the composite system is converted into a hybrid membrane, which is subsequently eluted to form a molecularly imprinted membrane [97]. Zhen et al. [73] reported a molecularly imprinted membrane based on a hybrid material. In this study, UiO-66@PDA nanoparticles were incorporated into Cellulose Acetate (CA) and PVDF to form a composite membrane (UPCPs). SiO_2_ was then introduced into the composite membrane to obtain a SiO_2_-modified UiO-66@PDA-CA-PVDF hybrid matrix nanoparticle composite membrane. The results indicate that the MOF nanoparticles in the base membrane, with their high specific surface area, significantly increased the density of imprinting sites; meanwhile, the introduction of inorganic SiO_2_ effectively enhanced adsorption efficiency by promoting interactions between functional monomers and template molecules. Furthermore, the synergistic effect of organic and inorganic materials significantly improved the anti-fouling performance of the imprinted membrane [74]. This study employed surface molecular imprinting technology, using KH-570-modified SiO_2_ nanoparticles as carriers, to construct molecularly imprinted nanoparticles with specific recognition sites on their surfaces via precipitation polymerization. The prepared imprinted nanoparticles were then loaded onto PVDF microfiltration membranes via vacuum-assisted self-assembly, resulting in a molecularly imprinted membrane with a hybrid material matrix. The introduced SiO_2_ components effectively enhanced the mechanical stability and structural integrity of the membrane material.

### 2.3. Classification Based on Mass Transfer Mechanisms

Based on differences in the mechanisms of molecular transport within the membrane [98,99], molecularly imprinted membranes can be classified into delayed permeation-dominated and facilitated permeation-dominated types.

#### 2.3.1. Delayed Permeation-Dominated MIMs

This class of molecularly imprinted membranes relies on specific adsorption as the core of selectivity and diffusion within the membrane as the rate-limiting step of mass transfer. Through the synergy between the adsorption selectivity of the imprinted sites and the diffusion control properties of the membrane structure, the target molecule, due to strong adsorption forces, is prevented from entering the pores or its diffusion is hindered, thereby achieving selective separation. Zhen et al. [100] reported an inorganic-organic hybrid molecularly imprinted membrane prepared via click chemistry. Through the synergistic activation and control of bifunctional groups, the formation of non-covalent bonds with ATSN molecules was facilitated, and the selective separation of ATSN was achieved by retarding its permeation during the permeation process. Furthermore, Sona et al. [101] successfully constructed polyvinylidene fluoride (PVDF)-based molecularly imprinted membranes using a phase-transition method and applied them to the removal of dyes from aqueous solutions. This study demonstrated that introducing functional fillers during the film-forming process enables synergy between the fillers and molecular imprinting technology, thereby enhancing the membrane material’s retention and removal efficiency for target pollutants.

#### 2.3.2. Permeation-Promoting MIMs

In contrast to delayed permeation-type membranes, permeation-enhanced molecularly imprinted membranes achieve high selectivity and high-throughput separation by constructing uniform pores within the membrane that match the size of the target molecules, while precisely distributing imprinted recognition sites complementary to the template molecules on the inner walls or surfaces of these pores. Target molecules pass through preferentially due to size matching and specific interactions, whereas non-target molecules are retained due to size mismatch or weak affinity. Chen et al. [102] reported a novel molecularly imprinted membrane and applied it to the selective separation of lysozyme. By studying the migration behavior of lysozyme on both imprinted and non-imprinted membranes, they confirmed that this molecularly imprinted membrane is a permeation-enhanced type. Gao et al. [67] developed a grafted molecularly imprinted membrane for the efficient separation of amino acids. Permeation experiments demonstrated that this imprinted membrane exhibits a dual-promotion mechanism for target molecules: first, template molecules are selectively adsorbed onto specific recognition sites on the membrane surface; subsequently, driven by the synergistic action of a concentration gradient and applied pressure, target molecules rapidly permeate through nanoscale channels within the membrane and diffuse into the receiving reservoir. This “adsorption-diffusion” synergy significantly enhances the permeation flux of target molecules, thereby achieving the goal of facilitated permeation separation.

## 3. Influence of the Physical Properties of MIMs on Selective Separation Performance

**Figure 2:**
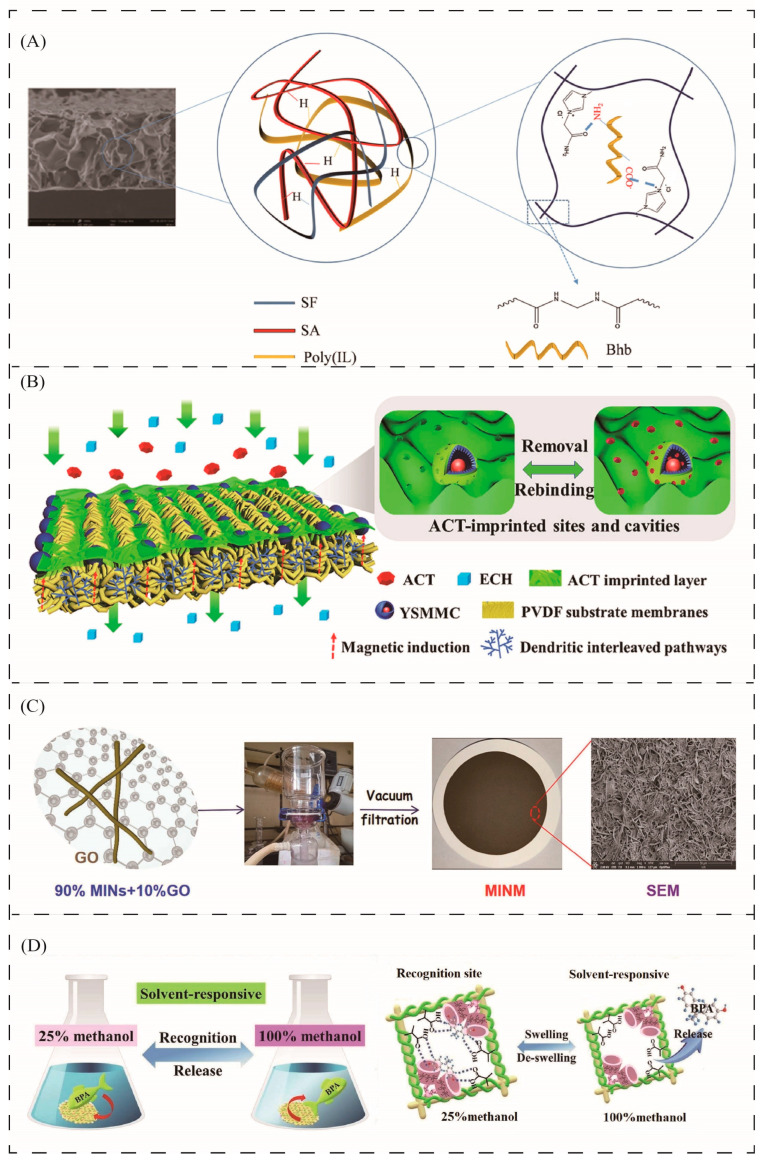
(**A**) Preparation of large-pore MIMs from SF/CA composites [103]. © 2022 Wiley Periodicals LLC. (**B**) Construction of instant noodle-like structures via magnetic guidance to increase specific surface area [104]. © 2024 Elsevier B.V. All rights are reserved, including those for text and data mining, Al training, and similar technologies. (**C**) Preparation of hydrophilic imprinted membranes using hydrophilic MnO_2_ nanowires and imprinted polymers [105]. © 2023 Elsevier B.V. All rights reserved. (**D**) Solvent-responsive MIMs that alter adsorption affinity through swelling [106]. © 2022 Elsevier B.V. All rights reserved.

The selective separation performance of molecularly imprinted membranes is the result of the synergistic interaction of their various physical properties. Figure 2 shows a schematic diagram illustrating the effects of different physical properties on selective separation.

### 3.1. Pore Size and Pore Structure

The recognition mechanism of molecularly imprinted membranes relies on the distribution of specific recognition sites within the membrane that are complementary to the template molecules in terms of shape, size, and functional groups [56]. If the pore size is too large, target molecules may pass through directly without binding to the imprinted sites, resulting in reduced selectivity; if the pore size is too small, target molecules may struggle to enter the channels and contact the imprinted sites, leading to low binding capacity and reduced flux. An appropriate pore size balances separation selectivity and flux. Molecularly imprinted membranes feature macropore, mesopore, and micropore channel structures. Macropores (pore size greater than 50 nm) serve as rapid mass transfer channels to reduce diffusion resistance and are typically used for the separation of proteins and macromolecules; mesopores (pore size between 2 nm and 50 nm) enhance selectivity while balancing flux and are typically used for drug separation and the removal of organic pollutants; microporous (pore size less than 2 nm) structures enable fine sieving and highly selective recognition, and are typically used for the separation of small molecules and gases [107,108].

In the design of membrane materials for the recognition of macromolecular proteins, the key challenge lies in constructing a superporous structure that combines high selectivity with efficient mass transfer capabilities. To this end, Fan et al. [103] successfully prepared a molecularly imprinted membrane with high recognition performance. The results indicate that, thanks to the synergistic effect of freeze-gel-assisted polymerization technology and the long-chain polymer of the 1-Vinyl-3-allylimidazolium Chloride([VAFMIM]Cl) functional monomer, the membrane formed a stable superporous structure. This unique pore structure not only effectively promotes the mass transfer of macromolecular proteins but also provides a favorable microenvironment for the specific adsorption of template molecules, significantly enhancing the membrane material’s recognition and separation efficiency for target proteins. Figure 2A shows a schematic diagram of the synthesis of this type of macroporous molecularly imprinted membrane. Additionally, Kashani et al. [109] systematically prepared a series of molecularly imprinted membranes using a phase-transition method and thoroughly investigated the regulatory effects of imprinted polymer loading on membrane structure and separation performance. By comparing the performance of the three membranes, MIM1, MIM2 and MIM3, the study found that as the content of the imprinting polymer increased, the pore size of the membrane first increased and then decreased; this may be attributed to the agglomeration effect of polymer particles under high loading. Notably, the MIM1 membrane with the smallest pore size (326 nm) exhibited the highest separation factor. The authors found that a larger average pore size may allow target molecules to easily penetrate the membrane layer, thereby weakening the membrane’s separation capacity.

Consequently, pore size optimization requires precise adjustment based on the size of the target molecules and the separation mechanism: for macromolecule recognition, ultra-large pores conducive to mass transfer should be constructed, whereas for small-molecule separation, excessively large pore sizes should be avoided to maintain high selectivity.

### 3.2. Specific Surface Area

Specific surface area is a key parameter influencing the adsorption capacity of the membrane, as it directly determines the maximum adsorption capacity of the molecularly imprinted membrane; the larger the specific surface area per unit mass of the imprinted membrane, the more adsorption sites it can provide [99,110]. At the same time, a high specific surface area enhances the mass transfer efficiency of the molecularly imprinted membrane, as a high specific surface area is typically accompanied by a porous structure. This facilitates the diffusion and mass transfer of target molecules within the membrane, thereby improving recognition efficiency and enhancing separation performance. Therefore, regulating the specific surface area can significantly improve the separation efficiency of molecularly imprinted membranes [111,112].

Chen et al. [104] reported a magnetically guided molecularly imprinted membrane filled with magnetically guided mesoporous carbon (YSMMC) for the effective separation of Acteoside(ACT). In this study, a large amount of YSMMC nanoparticle filler was concentrated on the upper surface of the membrane via an external magnetic field, followed by the construction of an imprinting layer on the membrane surface. Thanks to the magnetic-guided effect, YSMMC formed a unique “instant noodle-like” porous structure on the surface. Figure 2B shows a schematic diagram of this structure. characterization confirmed that this structure significantly increased the surface roughness and specific surface area of the membrane, thereby creating a highly efficient imprinting recognition interface. Another study on the separation of Atrazine (ATZ) explored the role of MOF nanocrystals. Xing et al. [65] successfully loaded the nanocrystals onto the surface of PVDF/Polyvinyl Alcohol (PVA) nanofiber membranes using a combination of electrospinning and solution back-diffusion, forming a “sticky bead” structure. Tests showed that these MOF sticky beads effectively improved the surface morphology of the nanofiber membranes and significantly increased their specific surface area. Permeation tests further confirmed the performance improvements resulting from structural optimization: compared to unmodified molecularly imprinted membranes, MOF-loaded membranes exhibited far superior re-binding capacity toward ATZ, while also contributing to enhanced permeation selectivity and extended operational lifetime during the selective permeation process. This demonstrates that constructing specific surface structures through the introduction of functional nanomaterials is an effective approach to enhancing the adsorption capacity and recognition efficiency of molecularly imprinted membranes.

### 3.3. Mechanical Strength

Mechanical strength refers to a membrane’s ability to resist deformation and rupture under pressure [113]. This is critical for practical applications (especially in high-pressure cross-flow filtration), as insufficient mechanical strength can lead to membrane structural collapse and reduced service life.

In practical applications, Wei et al. [114] synthesized the molecularly imprinted membrane D-MIM using a bulk imprinting method with ferulic acid and cinnamic acid as dual template molecules, and systematically investigated the effects of different types and dosages of nanoparticles (TiO_2_) on membrane performance. The results indicate that while the addition of inorganic nanoparticles generally enhances the mechanical strength of the membrane, it exerts significant size- and dosage-dependent effects on the permeability of template molecules and water flux. Through a comparative analysis of various nanocomposite membranes (such as 0.5–18 nmTiO_2_-D-MIM, 1–60 nm-TiO_2_-D-MIM), the study found that 1–18 nm-TiO_2_-D-MIM exhibited the best overall performance, achieving optimal separation efficiency and water flux while maintaining high mechanical strength. Furthermore, to address the issues of insufficient flexibility and low mechanical strength in molecularly imprinted membranes, the introduction of inorganic nanofillers for blending and modification has become an effective strategy. In another study [115], researchers successfully prepared performance-enhanced molecularly imprinted membranes by doping with SiO_2_ nanoparticles and systematically investigated the mechanisms by which nanoparticle size and concentration regulate the membrane’s comprehensive performance. The results indicated that 15 nm SiO_2_ particles exhibited the optimal modification effect, significantly enhancing the membrane’s mechanical strength while conferring the highest selectivity factor. Further investigation into the influence of nanoparticle concentration revealed that the membrane’s template binding capacity and water flux exhibited a trend of first increasing and then decreasing with rising SiO_2_ concentration, with 0.25 wt% identified as the optimal concentration threshold. Mechanistic analysis indicates that an appropriate amount of SiO_2_ nanoparticles facilitates the formation of functional monomer-imprinted complexes, thereby enhancing separation efficiency by increasing the number of effective imprinting sites on the membrane surface; however, when the nanoparticle concentration is too high, the particles tend to agglomerate, leading to a reduction in the number of imprinting sites and, consequently, a decline in separation performance.

In summary, the incorporation of inorganic nanofillers is an effective strategy for enhancing the mechanical strength of molecularly imprinted membranes. However, the size and concentration of nanoparticles exert a significant dual effect on membrane performance. For nano-composites prepared under optimal parameters, precise control of the physical parameters of the nanofillers is key to achieving a synergistic balance between high mechanical strength and high separation efficiency. This not only reinforces the mechanical strength of the membrane but also optimizes surface imprinting sites and mass transfer channels, thereby simultaneously improving separation efficiency and flux.

### 3.4. Hydrophilicity and Hydrophobicity

Hydrophilicity and hydrophobicity are important properties that influence a membrane’s fouling resistance [116], suitability for specific solvents (aqueous or organic systems) [117], and interactions with the molecules being separated (such as hydrophobic interactions) [118,119].

In a study on the highly selective separation of artemisinin, Meng et al. [105] innovatively synthesized a membrane by performing molecularly imprinted polymerization on MnO_2_ nanowires followed by vacuum filtration. Figure 2C shows a schematic diagram of the preparation of this hydrophilic membrane. The results indicated that the hydrophilic membrane surface primarily relies on specific functional group interactions and geometrically matched imprinted cavities to achieve precise recognition and separation of artemisinin. In contrast, the hydrophobic nanowire-based hydrophilic membranes primarily adsorb artemisinin through functional groups and matched imprinted pores, exhibiting high-specificity adsorption. In contrast, hydrophobic membranes demonstrate poor selectivity. This phenomenon is attributed to the hydrophobic environment, which tends to enhance non-specific interactions with the hydrophobic artemisinin ester, thereby weakening the membrane’s ability to specifically recognize the target molecule during competitive adsorption.

During the preparation of MIMs, the hydrophobicity of the membrane matrix often leads to non-selective adsorption of hydrophobic organic targets, thereby affecting separation selectivity. To address this issue, Ahmadi et al. [120] proposed an effective hydrophilic modification strategy. This study innovatively employed a novel bifunctional special-purpose monomer (GMA-β-CD) synthesized from glycidyl methacrylate-bonded β-cyclodextrin, combined with MBAA as a hydrophilic crosslinking agent. This design significantly enhanced the hydrophilicity of the imprinted membrane surface. This hydrophilic modification greatly suppresses the nonspecific adsorption of hydrophobic substances, thereby enhancing the membrane’s separation selectivity while ensuring the functionality of the specific recognition sites.

### 3.5. Swelling

The membrane absorbs solvent molecules in the solvent, causing volumetric expansion [121,122]. Excessive swelling alters the shape and size of the imprinted cavities, thereby reducing their selectivity. Therefore, a well-designed cross-linking structure and solvent compatibility are crucial for maintaining recognition performance [123].

In a study on the separation of bisphenol A in complex aqueous environments, Gao et al. [106] developed a molecularly imprinted membrane with solvent-responsive properties. The study ingeniously selected triethylene glycol dimethacrylate (TEGDMA) as the functional monomer; its core advantage lies in the flexible polyethylene glycol unit chains incorporated into its molecular structure, a structural feature that enables the membrane material’s solvent responsiveness. The performance was validated through swelling and deswelling cycles, and the results demonstrated that the imprinted membrane could effectively control the adsorption and desorption processes by adjusting the solvent ratio. Compared to traditional methods relying on conventional eluents, this strategy increased the desorption rate by more than 3.2 times, significantly improving separation efficiency and demonstrating immense potential and benefits for practical applications. Figure 2D shows a schematic diagram of a solvent-responsive molecularly imprinted membrane. Additionally, Liu et al. [124] successfully prepared a chitosan-based molecularly imprinted membrane. However, during the study, it was found that such membrane materials generally exhibit swelling in aqueous environments. This physical change directly leads to the contraction and deformation of the imprinted cavities, thereby reducing the number of specific recognition sites. To address this issue, the researchers investigated the influence of the solvent environment on membrane performance, particularly the regulatory effect of different ethanol volume fractions on swelling. Experimental results showed that as the ethanol content in the ethanol-water mixed solvent increased, the degree of membrane swelling decreased; in a 20% ethanol solution, the membrane morphology remained relatively stable. This study demonstrates that regulating the solvent ratio to suppress excessive membrane swelling is an effective strategy for enhancing the separation performance of chitosan-based molecularly imprinted membranes, providing an important reference for subsequent optimization of the membrane material’s resistance to swelling.

## 4. Strategies for Optimizing the Morphology of MIMs

### 4.1. Construction of Hierarchical Pore Networks

**Figure 3:**
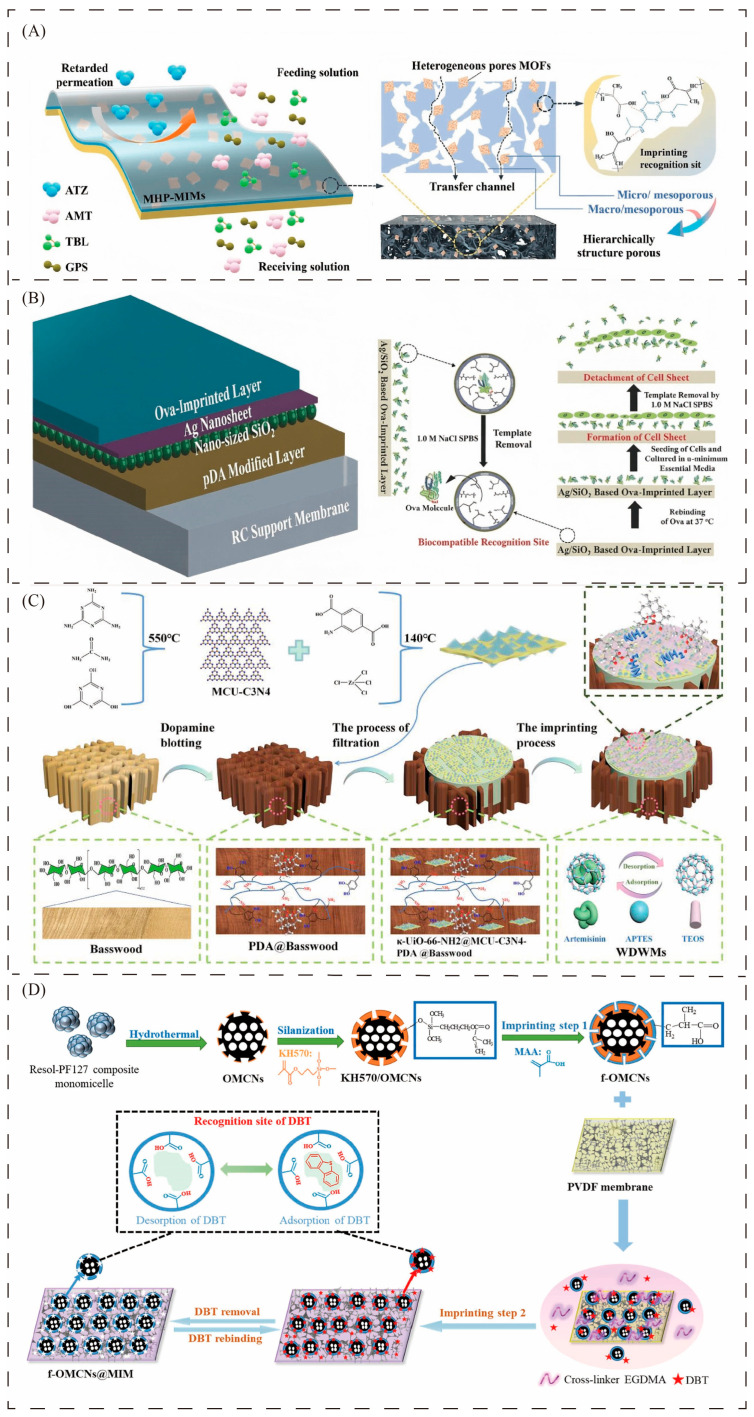
(**A**) A strategy for constructing hierarchical porous structures to enhance separation performance [128]. © 2025 Elsevier B.V. All rights are reserved, including those for text and data mining, Al training, and similar technologies. (**B**) Doping with SiO_2_/Ag nanoparticles to optimize the membrane’s overall performance [130]. © 2015 WILEY—VCH Verlag GmbH & Co. KGaA, Weinheim (**C**) Loading high-surface-area MOF nanomaterials onto the surface of the redwood membrane [131]. © 2023 Elsevier Ltd. All rights reserved. (**D**) Uniformly weaving porous carbon nanospheres (OMCNs) into the PVDF matrix to achieve an ordered distribution of imprint sites [132]. © 2021 Elsevier B.V. All rights reserved.

To enhance the synergistic effects of mass transfer and recognition, the design of hierarchical pore structures has become a major focus of current research [125,126]. Hierarchical pore structures feature a multi-level pore system comprising macropores, mesopores, and micropores. Macropores serve as rapid mass transfer channels to reduce diffusion resistance; mesopores carry imprinting sites to enhance selectivity while balancing flux; and micropores facilitate rapid adsorption and achieve high binding capacity [127]. Xing et al. [128] introduced MOF nanoparticles into a solidification bath to structurally modulate PVDF membranes, successfully preparing molecularly imprinted membranes with hierarchical porous structures. Figure 3A shows this layered porous structure. This method leverages the porous nature and interfacial interactions of MOF nanoparticles to induce the formation of a rich micro-/mesoporous structure, which not only significantly increases the membrane’s specific surface area and porosity but also provides a large number of active sites for the growth of the molecularly imprinted polymer. The resulting hierarchical porous structure facilitates the formation and exposure of more recognition sites, effectively ensuring the accessibility of the imprinted sites, while significantly reducing mass transfer resistance by optimizing pore connectivity. In another study, Lu et al. [129] reported a novel imprinted membrane based on a hierarchical microporous substrate. Using a thiol-ene click chemistry approach, the researchers successfully modified the substrate surface with a nanocomposite BPA molecularly imprinted layer featuring an irregular lattice distribution. This synergistic structural design combining a multi-level microporous substrate with an irregularly arrayed polymer layer offers significant advantages: the multi-level microporous substrate provides excellent mass transfer channels, while the irregularly arrayed polymer layer offers a high density of active recognition sites. The synergistic interaction between the two effectively enhances the separation performance of the imprinted membrane, improves the recognition capacity for the target molecule (bisphenol A), and optimizes mass transfer kinetics.

### 4.2. Nanomaterial Composite Reinforcement

To synergistically optimize the microstructure and comprehensive performance of imprinted membranes, the incorporation of functional nanomaterials has emerged as an effective enhancement strategy [133]. This approach endows the membrane material with various new properties by doping or modifying the membrane structure with inorganic or metallic nanoparticles [134,135].

The introduction of inorganic nanoparticles such as SiO_2_, TiO_2_, and Fe_3_O_4_ can regulate the phase separation behavior during the formation of imprinted membranes, as well as their pore structure, hydrophilicity/hydrophobicity, and anti-fouling properties. It can also induce the formation of hierarchical pores, thereby enhancing mechanical strength and thermal stability. For example, Wu et al. [130] successfully constructed SiO_2_/Ag nanocomposite imprint membranes on a mussel-derived porous substrate. Figure 3B illustrates the composite structure of this membrane. By performing protein imprinting polymerization on the nanomembrane, they not only achieved highly selective recognition of the target protein but, more importantly, the doping of SiO_2_ and Ag nanoparticles significantly optimized the membrane’s overall performance. The results indicate that this composite structure not only effectively enhances the antibacterial properties of the imprinting membrane and prevents biological contamination but also significantly improves permeation flux. This strategy provides a new technical approach to addressing contamination and efficiency issues in the separation of biomacromolecules while maintaining excellent biocompatibility, demonstrating application potential in the separation and purification of complex biological samples.

Similarly, the incorporation of materials such as carbon nanotubes, graphene, and MXene facilitates the formation of numerous multi-level pore channels in the imprinting membrane, thereby enhancing mass transfer and flux. Furthermore, the inherent strength of these materials improves mechanical properties and thermal stability, while surface-functionalized materials can also enhance hydrogen bonding or electrostatic interactions at the imprinting sites; Taking MXene as an example, Bacal et al. [136] combined MXene nanosheets with a polymer support membrane and constructed a molecularly imprinted layer on its surface. Studies have shown that this strategy not only significantly improves the mechanical properties of the imprinted membrane but also that the incorporated MXene enhances surface reactivity and biocompatibility. Furthermore, the presence of MXene can effectively optimize the polymerization process. In summary, these synergistic effects confer broad development prospects for MXene-enhanced imprinting membranes in biotechnology fields such as biological separation.

### 4.3. Surface Topography Design

This design, through the use of nanoscale protrusions, microsphere aggregates, columnar arrays, and wrinkled surfaces, can increase membrane surface roughness and substantially enhance the specific surface area. Furthermore, this morphological control strategy increases the exposure and accessibility of imprinting sites, shortens mass transfer pathways, and reduces transmembrane resistance, ultimately achieving a synergistic improvement in both selectivity and flux. For example, in MOF-based composite molecularly imprinted membranes, MOFs provide inherent micropores, mesopores, and high specific surface area; through in situ growth or doping and blending, a “MOF core-imprint” multi-level structure is constructed; Yan et al. [131] innovatively loaded MOF materials onto the surface of dopamine-modified redwood membranes. Figure 3C is a schematic diagram of this modification method. By leveraging the high specific surface area and structural tunability of MOF nanoparticles, they provided a rich, irregular lattice structure for the formation of recognition sites. Experimental results demonstrate that the imprinted membranes prepared using this strategy exhibit excellent selective recognition capabilities, efficient rebinding performance, and high-throughput transport characteristics, offering a new approach to interface design for the development of high-performance, structurally controllable molecularly imprinted membranes.

### 4.4. Synergistic Optimization of the Preparation Process

The preparation process is a critical factor in determining the microstructure and functional properties of molecularly imprinted membranes. Dynamically adjusting key process parameters to achieve a precise match between membrane structure and function is an important research direction for enhancing the overall performance of imprinted membranes.

In the field of electrospinning technology, systematic optimization of key parameters such as voltage and flow rate can effectively control the fiber morphology and surface roughness of the base membrane. Su et al. [100] employed coaxial electrospinning technology to prepare molecularly imprinted fiber membranes with a rough surface structure. This study utilized coaxial electrospinning to independently regulate the concentration ratios of dual crosslinkers (1,4-Butanediol diglycidyl ether and tetrakis phosphonium chloride) in the core and shell streams, thereby achieving precise control over the microstructure of the fiber membrane. This dual-fluid synergistic crosslinking strategy not only endows the fiber membrane with excellent mechanical strength but also constructs an ideal surface morphology, thereby providing a structural foundation for enhancing the accessibility of imprinting sites and adsorption kinetics and demonstrating an effective pathway to achieve multifunctional material design through process parameter optimization. On the other hand, the pre-design of the base membrane pore size is also a key approach to optimizing the performance of imprinted membranes. During the preparation process using the phase inversion method, the addition of pore-forming agents can precisely regulate the ratio of macropores to mesopores in base membranes such as PVDF; based on this mechanism, Huang et al. [137] successfully prepared a molecularly imprinted membrane for kaempferol using the phase inversion method and introduced inorganic salts as pore-forming regulators to control the membrane’s microstructure. The study found that the type and concentration of inorganic salts exert significant differential effects on the membrane’s pore size and porosity. This mechanism primarily stems from the interference of inorganic salts with the thermodynamic and kinetic behavior of the polymer solution during the phase inversion process, thereby influencing the rate of phase separation. Therefore, by precisely controlling the amount of inorganic salt (pore-forming agent) added, it is possible to achieve targeted optimization of the pore structure of the imprinted membrane. This enhances the membrane’s permeability and adsorption capacity while ensuring specific recognition capabilities, providing an important process control method for the preparation of high-performance porous imprinted separation membranes.

### 4.5. Construction of Ordered and Regularly Arranged Membranes

In the preparation of molecularly imprinted membranes, achieving an ordered arrangement and regular distribution of functional sites is critical to determining their recognition selectivity and mass transfer efficiency. Conventional bulk polymerization often results in imprinting cavities that are buried too deeply, leading to high mass transfer resistance and the presence of non-selective adsorption sites. Therefore, constructing an imprinting layer with a regular structure and high accessibility has become one of the core strategies for enhancing membrane performance. Against this backdrop, Qu et al. [132] proposed a stepwise imprinting strategy based on porous carbon nanospheres (OMCNs). Figure 3D shows a schematic illustration of this imprinting strategy. By pre-grafting functional monomers onto the OMCNs’ surface, they successfully constructed rich and uniform dibenzothiophene (DBT) imprinting cavities. These functionalized nanospheres were then uniformly woven into a PVDF matrix, achieving an ordered distribution of imprinting sites within the membrane, thereby significantly enhancing the membrane’s rebinding capacity and selective recognition performance toward target molecules. In another study, Sun et al. [138] innovatively introduced nano-molecularly imprinted polymers (MIPs) into a breath-forming (BF) system, using MIPs as functional templates to prepare molecularly imprinted membranes with highly ordered microporous structures. The results indicate that the introduction of MIP not only provides specific recognition sites but its unique surface properties also help induce the self-assembly of water droplet templates, thereby significantly promoting the formation of an ordered porous morphology on the membrane surface. This membrane material, characterized by the synergy of structure and function, has successfully achieved simultaneous improvements in adsorption capacity, membrane flux, and selectivity.

## 5. Separation Applications of Novel MIMs

**Figure 4:**
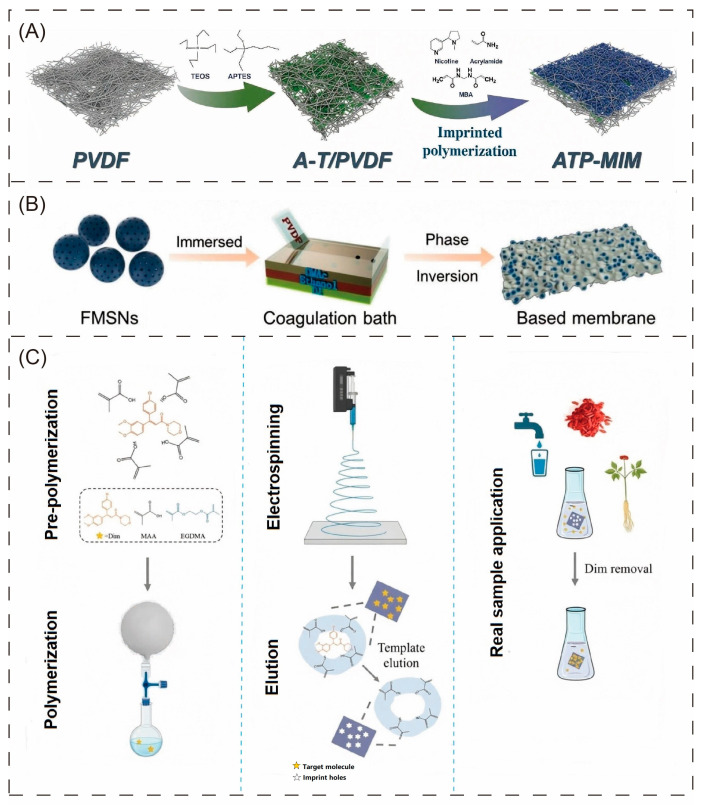
(**A**) Surface modification of PVDF membranes using APTES and TEOS [143]. © 2025 Elsevier B.V. All rights are reserved, including those for text and data mining, Al training, and similar technologies. (**B**) Incorporation of FMSNs into PVDF membranes [144]. © 2026 Published by Elsevier Ltd. (**C**) Simultaneous polymerization and film formation via electrospinning [145]. © 2026 Elsevier B.V. All rights are reserved, including those for text and data mining, Al training, and similar technologies.

In recent years, MIMs have demonstrated immense application potential in the separation and purification of complex systems due to their highly specific recognition of target molecules [139,140,141,142]. Table 2 shows the applications of MIMs in the field of separation in recent years. Through innovative material design and preparation processes, researchers have successfully developed a variety of high-performance imprinted membranes. Figure 4 shows a variety of high-performance imprinting films developed in recent years.

**Table 2:** A Comparison of MIMs Separation Applications.

Membrane	Membrane Matrix	Physical Characteristics	Separation Applications	Ref.
Hemodialysis Imprinted Fiber Membrane	Polysulfone	High mechanical properties and hydrophilicity	Biomedical Science, Hemodialysis	[146]
Nicotine-Specific Molecularly Imprinted Adsorption Membrane	Polyvinylidene fluoride	High specific surface area, surface functionalization	Biomedical Science, Drug Separation	[143]
Nano-composite imprinting membrane	Polyvinylidene fluoride, silicon dioxide	High specific surface area	Water Pollution Control	[144]
Electrospun Molecularly Imprinted Membranes	Acrylonitrile	Integrated Manufacturing Process, Mechanical Durability	Environmental Pollution Control	[145]

In the biomedical field, significant progress has been made in developing specialized imprinted membranes for hemodialysis and drug separation. Djunaidi et al. [146] (2026) used polysulfone as a matrix and employed a phase-transition method to prepare an imprinted fiber membrane for hemodialysis. Through a carrier functionalization strategy, this study not only endowed the membrane with excellent mechanical properties but also significantly enhanced its hydrophilicity and permeability selectivity, enabling it to efficiently separate uremic toxins and demonstrating great potential as a next-generation hemodialysis material. Similarly, Tang et al. [143] reported a polyvinylidene fluoride (PVDF)-based molecularly imprinted membrane for nicotine separation. This study innovatively utilized 3-Aminopropyltriethoxysilane (APTES) and Tetraethyl Orthosilicate (TEOS) to surface-modify the PVDF membrane, forming a coating rich in amino functional groups and featuring a rough surface. This coating not only provided robust anchoring sites for the imprinting layer but also synergized with it to achieve strong adsorption capacity and high permeability selectivity for nicotine.

Regarding nano-composite imprinted membranes, the introduction of inorganic nanomaterials has opened new avenues for enhancing membrane recognition performance. Yu et al. [144] successfully constructed a highly efficient molecular recognition system by introducing mesoporous silica nanoparticles (FMSNs) into a solidification bath and precisely regulating the solvent ratio to control the distribution of nanoparticles on the PVDF substrate. In this system, FMSNs serve as scaffolds for recognition sites, not only increasing the membrane’s specific surface area but also providing abundant binding sites for target molecules, thereby determining the membrane’s high selectivity toward the target.

In the field of environmental pollutant remediation, highly efficient remediation technologies for pesticide residues have long been a research focus. Li et al. [145] innovatively proposed an integrated preparation strategy and successfully constructed a polyacrylonitrile (PAN)-based molecularly imprinted membrane for the efficient removal of dimethomorph(Dim) from the environment. This study ingeniously utilized electrospinning technology to achieve simultaneous completion of the polymerization reaction and film formation. This integrated process not only significantly simplifies the preparation workflow and reduces production costs but also endows the membrane material with excellent mechanical durability. Experimental results demonstrate that the imprinted membrane exhibits extremely high removal efficiency for the target pesticide Dim. This research provides a highly promising, scalable, and economically viable solution for the remediation of pesticide pollutants in environmental water bodies, holding significant implications for advancing the practical application of molecularly imprinted technology (MIT) in the field of environmental remediation.

## 6. Conclusions

Although MIMs have achieved significant results in laboratory research, their large-scale industrial application still faces numerous challenges. The focus of this review is to elucidate the intrinsic relationship between the microstructure of MIMs and their macroscopic separation performance. The spatial distribution of imprinted sites is the primary factor influencing MIM’s performance. Compared to monolithic imprinted membranes, in which recognition sites are deeply embedded within the membrane matrix, surface-imprinted membranes and composite membranes significantly shorten the mass transfer path for target molecules by exposing the recognition sites to the membrane surface or near-surface regions. This effectively addresses the key bottlenecks of high mass transfer resistance and poor site accessibility in traditional MIMs, thereby achieving higher flux and faster adsorption kinetics.

The separation performance of MIMs is the result of the synergistic interaction of their various physical properties. Pore size must be precisely matched to the size of the target molecules to strike a balance between selectivity and flux. Constructing a multi-level pore structure comprising macro-, meso-, and micro-pores is an ideal strategy, where macro-pores serve as rapid mass transfer channels, while meso- and micro-pores host the imprinted sites to enable highly selective recognition. A high specific surface area provides the foundation for loading more imprinted sites and directly determines the membrane’s adsorption capacity. Introducing nanomaterials or constructing specialized surface topologies are effective means of increasing the specific surface area. Good mechanical strength is a prerequisite for the membrane to maintain structural integrity and long-term stability under high-pressure operation. Swellability, however, is a double-edged sword; excessive swelling can disrupt the geometric configuration of the imprinted cavities, leading to a decrease in selectivity. Therefore, it is crucial to enhance the membrane’s dimensional stability by optimizing the degree of cross-linking or introducing rigid nanofillers. The hydrophilicity or hydrophobicity of the membrane not only affects its applicability in different solvent systems but also directly relates to its fouling resistance. Hydrophilic surfaces can effectively suppress the nonspecific adsorption of hydrophobic contaminants, thereby maintaining high selectivity in complex systems.

To advance this field from theory to practice, future research should focus on the following directions.

Current MIMs preparation methods often involve cumbersome steps and rely on large amounts of organic solvents. There is an urgent need to develop scalable, low-cost, and environmentally friendly preparation processes. For example, exploring aqueous polymerization systems, utilizing supercritical CO_2_ as a green solvent, or developing one-step forming technologies (such as combining polymerization with electrospinning) can reduce process steps and solvent consumption, laying the foundation for the industrial production of MIMs.

When treating complex real-world samples such as blood and industrial wastewater, membrane fouling is the primary cause of performance degradation. Future research should go beyond simple hydrophilic modification and focus on developing smart MIMs with active anti-fouling capabilities. For example, designing membrane materials with zwitterionic surfaces, photocatalytic self-cleaning properties (such as utilizing the photocatalytic properties of MOFs to degrade pollutants), or enzyme-responsive characteristics to enable long-term stable operation in complex matrices.

Most research remains at the stage of validation using simulated solutions. To demonstrate the practical application value of MIMs, separation tests must be conducted on real, complex samples (such as untreated industrial wastewater, bioreactor effluent, and plasma). This will comprehensively evaluate the MIMs’ resistance to interference, stability, and regenerative performance and is an essential step in bridging the gap between laboratory research and practical application.

One of the greatest advantages of membrane technology lies in its ease of continuous and automated operation. Future research should focus on integrating high-performance MIMs with existing continuous separation systems (such as membrane chromatography and continuous membrane filtration units). Exploring MIMs’ packing methods, hydrodynamic behavior, online regeneration strategies, and long-term operational stability within these systems will significantly advance the practical application of MIMs in fields such as pharmaceuticals, bioengineering, and fine chemicals.

## Figures and Tables

**Table 1:** A Comparison of Different Categories of MIMs.

Membrane Classification	Characteristics	Applications	Refs.
Bulk-imprinted membranes	This method results in a membrane with a continuous, non-porous, or porous monolithic structure, with specific recognition sites uniformly embedded within the membrane matrix	Achieves high binding capacity and rapid mass transfer through a continuous three-dimensional network structure, making it suitable for the separation of biomacromolecules	[64,65]
Surface-imprinted membranes	This method forms a thin, specific recognition layer on the surface of the support substrate, with recognition sites distributed only on or near the membrane surface	The recognition sites are primarily concentrated in the surface layer of the membrane, which significantly shortens the mass transfer path, making it suitable for the targeted separation of specific substances in complex aqueous systems.	[66,67]
Composite membranes	This method is simple to implement and cost-effective; by adjusting the proportion and particle size of the imprinting material, the membrane’s selectivity, flux, and mechanical stability can be flexibly controlled	Physical blending balances the performance and cost of different materials, making it suitable for applications that require both mechanical strength and functionalization.	[68,69]
Organic polymer-based MIMs	This membrane is primarily composed of organic polymer materials, with organic or natural polymers serving as the base membrane	It has excellent chemical stability and is suitable for the separation of toxins in complex food systems.	[70,71]
Inorganic-based MIMs	Inorganic-based molecularly imprinted membranes using inorganic materials as the core matrix or support	Resistant to high temperatures, strong acids and alkalis, and organic solvents; suitable for the resource recovery of industrial waste liquids	[72]
Hybrid material-based MIMs	This type of membrane uses an organic polymer as its main framework and incorporates inorganic nano-functional phases as functional units	Combining a high specific surface area with specific recognition capabilities, it is suitable for challenging chiral separations.	[73,74]

## Data Availability

Data is contained within the article.

## Abbreviations

The following abbreviations are used in this manuscript:

MIMs	molecularly imprinted membranes
PVDF	polyvinylidene fluoride
NC	cellulose
PCL	polycaprolactone
GO	graphene oxide
CA	cellulose acetate
ACT	acteoside
ATZ	atrazine
PVA	polyvinyl alcohol
TEGDMA	triethylene glycol dimethacrylate
DBT	dibenzothiophene
MIPs	molecularly imprinted polymers
BF	breath-forming
APTES	3-aminopropyltriethoxysilane
TEOS	tetraethyl orthosilicate
PAN	polyacrylonitrile
Dim	dimethomorph
MIT	molecularly imprinted technology
